# A versatile quantum walk resonator with bright classical light

**DOI:** 10.1371/journal.pone.0214891

**Published:** 2019-04-09

**Authors:** Bereneice Sephton, Angela Dudley, Gianluca Ruffato, Filippo Romanato, Lorenzo Marrucci, Miles Padgett, Sandeep Goyal, Filippus Roux, Thomas Konrad, Andrew Forbes

**Affiliations:** 1 School of Physics, University of the Witwatersrand, Private Bag 3, Wits 2050, South Africa; 2 CSIR National Laser Centre, PO Box 395, Pretoria, South Africa; 3 Department of Physics and Astronomy G. Galilei, University of Padova, Padova, Italy; 4 CNR-INFM TASC IOM National Laboratory, Trieste, Italy; 5 Dipartimento di Fisica, University di Napoli Federico II, Complesso Universitario di Monte S. Angelo, Napoli, Italy; 6 SUPA, School of Physics and Astronomy, University of Glasgow, Glasgow, United Kingdom; 7 Indian Institute of Science Education and Research, Mohali, Punjab, India; 8 National Metrology Institute of South Africa, Pretoria, South Africa; 9 School of Chemistry and Physics, University of KwaZulu-Natal, Durban, South Africa; University of the Basque Country, SPAIN

## Abstract

In a Quantum Walk (QW) the “walker” follows all possible paths at once through the principle of quantum superposition, differentiating itself from classical random walks where one random path is taken at a time. This facilitates the searching of problem solution spaces faster than with classical random walks, and holds promise for advances in dynamical quantum simulation, biological process modelling and quantum computation. Here we employ a versatile and scalable resonator configuration to realise quantum walks with bright classical light. We experimentally demonstrate the versatility of our approach by implementing a variety of QWs, all with the same experimental platform, while the use of a resonator allows for an arbitrary number of steps without scaling the number of optics. This paves the way for future QW implementations with spatial modes of light in free-space that are both versatile and scalable.

## Introduction

Quantum walks (QWs) may arguably be dated back to Feynman and even Dirac [1], but certainly have become topical since the seminal paper by Aharonov et. al. [2], offering a probability distribution that spreads quadratically faster than that of a classical random walk. In a typical random walk a discrete walker is randomly moved in directions, say left or right, indicated by the outcome of a random value generator (coin toss). Unlike in the case of the classical random walk, a superposition of coin and position states occur for all possibilities in the QW, that is, all possible paths are walked at each step in the process. This changes the dynamics of propagation, resulting in interference of the probability amplitudes for each position held by the walker and inducing a ballistic speedup in the probability distribution variance over the classical equivalent. The interest in QWs stems partly from the realisation that they provide a universal basis for quantum computation [3], presenting an alternate route for successful development of a feasible quantum computer. While single-particle QWs do not yield significant computational advantage over classical computation (resources scale exponentially with equivalent encoded qubits), multiple interacting [4] and non-interacting [5] walkers do, as would multiple dimensions with a single particle. Consequently, a large variety of QW algorithms have been designed, allowing database searching [6, 7], navigation of networks [8], quantum simulation [9–11] and element distinctness determination, [12] among others. Recently, its application has been extended into quantum cryptography with image encryption [13] and quantum key distribution [14] protocols. Studies into photosynthetic energy transport in systems also show the natural occurrence of QWs [15], indicating its promise as a modeling technique for certain natural processes.

Inevitably there has been much attention on how to actually implement a QW, which have now been demonstrated with many systems including Nuclear Magnetic Resonance (NMR) [16], electrons [17], atoms [18], ions [19, 20], photons [10], Bose-Einstein condensate [21], optical fiber time loops [22], OAM [10], photonic waveguide arrays [23, 24], beam displacers [25] and cascaded q-plates [26, 27], all in the quantum regime. Several authors have also made use of attenuated classical light to implement QWs. A distinct difference between implementations here is that measurement does not collapse the wavefuncion into a singular value, which is necessary for some applications such as random number generators [28]. In some instances, a discrete outcome may, however, be achieved through attenuation of the light before detection. Such set-ups have included optical cavities [29], photonic crystal chips [30], and optical fiber time loops [31, 32]. In these approaches the walker is directed through physical paths involving multiple interferometers to achieve the interference effect, without the use of a coin degree of freedom, akin to a Galton board. Here cascaded q-plates [33] have also been employed, avoiding this issue, however still subject to scalability and measurement issues. Additionally, free-space QWs have been demonstrated by tailoring the wavefunction to interfere with propagation [34, 35].

**Fig 1:**
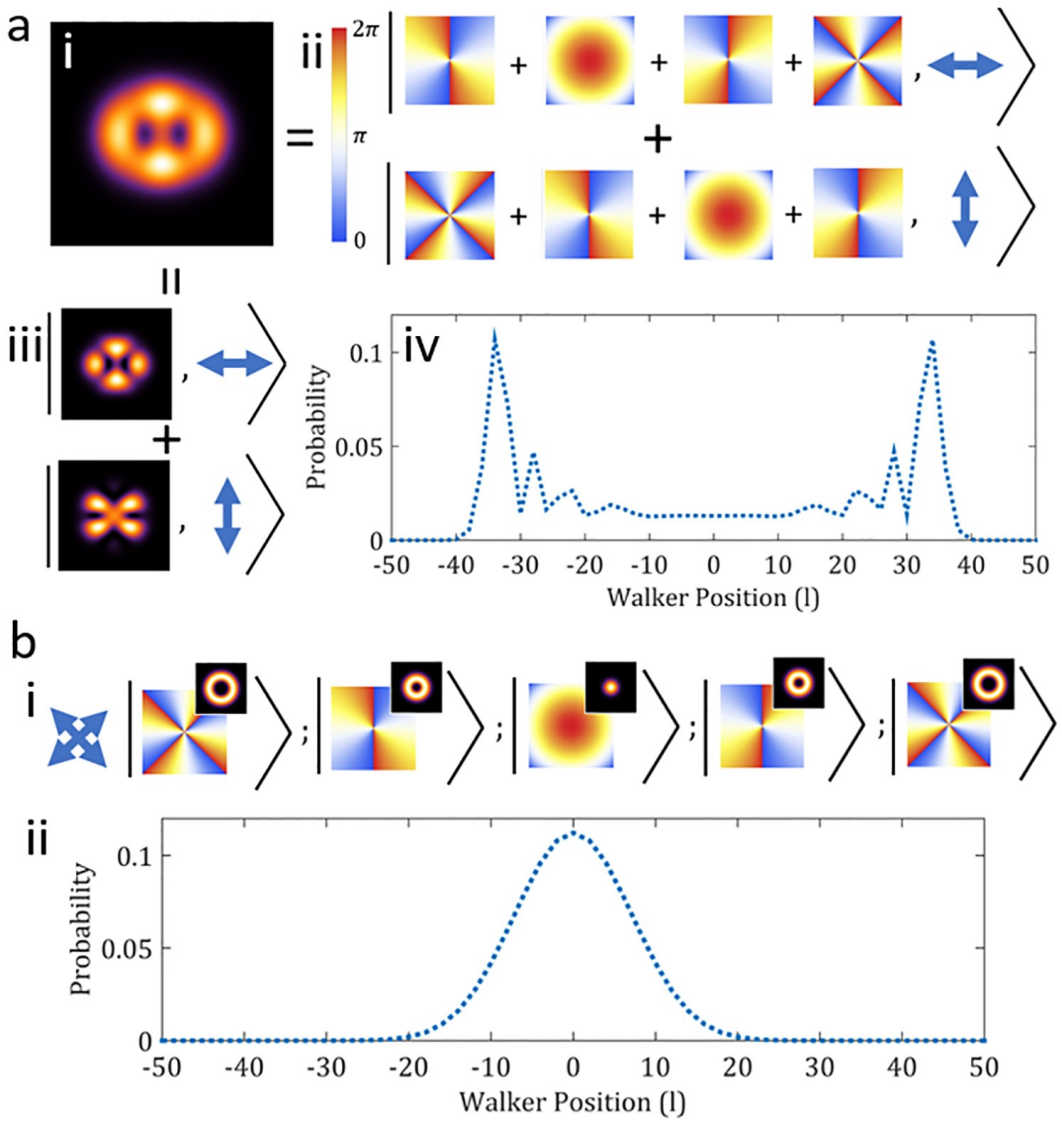
Classically entangled light. Illustration of the QW reliance upon the non-separability of OAM and polarization states of classical light. (a) shows the non-separable superposition of OAM states and polarization for a 4 step QW. False color density plots (ii) show the phase distribution of the OAM modes *l* = {-4, -2, 0, 2, 4}, forming the positions occupied by the walker (light beam). Weighted superpositions of these would then yield the spatial modes shown in (iii) with the associated polarization states. Together, the spatial distribution in (i) is formed, where the QW is contained in a single beam of classically entangled light. After many steps, the walker occupies positions near the end of the distribution with greater probability as is shown for 50 steps of a symmetrical walk (iv). (b) shows the 4-step classical RW analogue where the walker may only assume one position (OAM) state at a time. Here, the state of the walker remains separable. Accordingly, the light beam (walker) contains only one of the possible OAM states shown as with different kets and a separable polarization state (i). Insets show the spatial distribution of the beam for each case. (ii) measurements of the repeated walk then results in a probability distribution after 50 steps where the walker occupies positions at the center with the greatest probability.

Using a coin makes it possible to implement QWs without interferometers. For this purpose, entanglement (non-separable correlation) between the coin and position degrees-of-freedom (DoF) is a necessary requirement. In this sense, coined QWs may be considered to act as entanglement generators [36], and the use of classical light to achieve this has been outlined theoretically [37]. But non-separability is not unique to quantum mechanics and has been observed in many classical systems, including the non-separable correlations in local DoFs of vector beams [38–41]. These correlations are known as “classical non-separability” or “classical entanglement” [42]. Fig 1 illustrates this concept, contrasting the difference between (a) exploiting non-separability to yield a QW as opposed to (b) when light is allowed to evolve without manipulation of the non-separability. Consequently a classical RW occurs.

Here we experimentally demonstrate a versatile and scalable resonator configuration, as proposed by Ref. [37], to realise quantum walks with bright classically entangled light. We use vector vortex beams and geometric phase to demonstrate a quantum walk that takes place with spatial modes of classical light in orbital angular momentum (OAM) space where polarization acts as the internal coin state. The versatility of our approach allows us to implement a variety of QWs, for example, symmetric and asymmetric QWs, transitions from a Hadamard coin to a balanced coin QW and a NOT-coin QW, all with the same experimental platform. Our resonator-type configuration, which is implemented by means of a ring cavity, overcomes scalability and flexibility issues associated with many cascaded step schemes, where the resources scale linearly with the number of steps.

## Results

### Theory

Like in a classical random walk, each step of a quantum walk consist of a change of the coin state (corresponding to a coin toss in a random walk) and a shift of the position of a walker according to the coin state. Our discrete time quantum walk of *n* steps on a one-dimensional lattice requires a system with two degrees of freedom. The first degree of freedom (*DoF*) has to be associated with a linear state space that comprises a number 2*n* + 1 of orthogonal states and the second DoF needs at least two orthogonal states. In our case we choose a laser pulse with Gaussian profile carrying OAM (*l*) that can assume the values *l* = −*n*, −*n* + 1, …*n*. It is well known that the corresponding optical Laguerre Gauss modes correspond to elements of a Hilbert space of square-integrable functions and can be represented in Dirac notation by the vectors |*l* = −*n*〉, |*l* = −*n* − 1〉, …|*l* = *n*〉. The polarisation of the light corresponds to the second degree of freedom spanned by left-circular and right circular basis states, represented by the Dirac ‘ket vectors’ |*L*〉 and |*R*〉, respectively. Here we consider the states from the point of view of the source.

A move of the walker to the left or right, given that the coin shows the value *L* or *R*, respectively, can then be expressed by a shift operator $\hat{S}$ acting on the OAM and polarisation states of the light pulse:

$$\hat{S}=\sum_{l=-\infty }^{\infty }|l+2q\rangle \langle l|⊗|L\rangle \langle R|+|l-2q\rangle \langle l|⊗|R\rangle \langle L|.$$ (1)

This addition and subtraction of the value 2*q* depending on the polarisation, can be realised by using a so-called q-plate (cp. description of the experiment) where the desired polarisation dependant OAM is generated with geometric phase. This then corresponds to a translation (*‘walk’*) in OAM space.

Note that the action of the q-plate described by the shift operator $\hat{S}$ includes an additional flip of the polarisation, which corresponds to a change of the coin state. This effect has to be taken into account when defining the coin toss operator. We implemented several coin toss operators (see below). All of these can be easily realised by means of quarter-wave plates (QWP),

$$\begin{array}{c} {\hat{Q}}_{\theta }=\frac{1}{\sqrt{2}}(|R\rangle \langle R|+i\text{exp}(-i2\theta )|R\rangle \langle L|\\ +i\text{exp}(i2\theta )|L\rangle \langle R|+|L\rangle \langle L|)\;, \end{array}$$ (2)

and half-wave plates (HWP),

$${\hat{H}}_{\theta }=i\text{exp}\left(-i2\theta \right)|R\rangle \langle L|+i\text{exp}\left(i2\theta \right)|L\rangle \langle R|\;,$$ (3)

where *θ* is the angle of the fast axis of the plates to the horiziontal axis. For example, a QWP with a fast-axis angle of 45° combined with a q-plate results in a QW with a Hadamard coin specified by the propagation operator

$$\begin{array}{c} {\hat{Z}}_{H}\equiv {\hat{Q}}_{45^{\circ }}\hat{S}=\sum_{l}(|l+2q\rangle \langle l|⊗\frac{1}{\sqrt{2}}\left(|R\rangle +|L\rangle \right)\langle R|\\ +|l-2q\rangle \langle l|⊗\frac{1}{\sqrt{2}}\left(|R\rangle -|L\rangle \right)\langle L|)\;. \end{array}$$ (4)

More details are provided in the Supplementary Information. Here the propagator *Z*_*H*_ couples OAM (walker) to the polarisation (coin), i.e. it generates a vector beam from a scalar beam. The non-separability of the polarisation and the spatial degrees of freedom in the vector beam corresponds to the quantum entanglement between the walker and the coin degrees of freedom in realisations of the quantum walk with quantum systems.

Choosing the polarisation to represent the coin state has the advantage that all coin toss operators $\hat{C}$, which form the group SU(2) of special unitary operators, can be simply realised by a combination of at most two quarter-wave plates and a half-wave plate [43]. Moreover, it can be shown that the three parameters (the so-called Euler angles) that characterise any coin toss operator *C* ∈ SU(2) can be reduced by local basis transformations on the coin and the walker space to a single parameter that distinguishes the dynamics of all quantum walks with time-independent coin toss operator [44]. As a result, any quantum walk can be expressed by a propagator of the form.

$${\hat{Z}}_{\theta }={\hat{C}}_{\theta }{\hat{C}}_{N}\hat{S}\;,$$ (5)

where ${\hat{C}}_{\theta }$ has the matrix representation

$${\hat{C}}_{\theta }=\frac{1}{\sqrt{2}}[\begin{array}{cc} \text{cos}\frac{\theta }{2} & i\;\text{sin}\frac{\theta }{2}\\ i\;\text{sin}\frac{\theta }{2} & \text{cos}\frac{\theta }{2} \end{array}],$$ (6)

with respect to the basis of circular polarisations |*R*〉, |*L*〉. We have inserted a polarisation flip *C*_*N*_ (also known as *Not Coin*, see below) to compensate the flip introduced by the q-plate. In this way the coin operators coincide with those conventionally used for quantum walks. The combination ${\hat{C}}_{\theta }{\hat{C}}_{N}$ can be implemented using two quarter-wave plates $\hat{Q}$ and one half-wave plate $\hat{H}$:

$${\hat{C}}_{\theta }{\hat{C}}_{N}={\hat{Q}}_{45^{\circ }}{\hat{H}}_{\frac{\theta }{4}}{\hat{Q}}_{45^{\circ }}\;,$$ (7)

where we have suppressed an irrelevant global phase factor.

After *n* steps with propagator $\hat{Z}=\hat{C}\hat{C_{N}}\hat{S}$ the initial state |*ψ*〉_0_ of the walker will have evolved to the state |*ψ*〉_*n*_ with

$$|{\psi \rangle }_{n}={\hat{Z}}^{n}{|\psi \rangle }_{0},$$ (8)

Our approach makes it possible to study all QW configurations with time-independent coin. By way of example, we implemented the usual QW with a Hadamard coin

$${\hat{C}}_{H}=\frac{1}{\sqrt{2}}[\begin{array}{cc} 1 & 1\\ 1 & -1 \end{array}],$$ (9)

as well as a balanced coin

$${\hat{C}}_{B}=\frac{1}{\sqrt{2}}[\begin{array}{cc} 1 & i\\ i & 1 \end{array}],$$ (10)

a NOT coin

$${\hat{C}}_{N}=[\begin{array}{cc} 0 & 1\\ 1 & 0 \end{array}],$$ (11)

and an Identity coin

$${\hat{C}}_{I}=[\begin{array}{cc} \;1 & 0\\ 0 & 1 \end{array}].$$ (12)

Demonstrating the versatility of the proposed setup, each of these coins could be realised with a single wave plate: $\hat{C_{H}}\hat{C_{N}}={\hat{Q}}_{45^{\circ }}$, $\hat{C_{B}}\hat{C_{N}}={\hat{H}}_{90^{\circ }}$, $\hat{C_{I}}\hat{C_{N}}={\hat{H}}_{0^{\circ }}$, and the NOT coin ${\hat{C}}_{N}$ without wave plate since it is already included in the action of the q-plate.

### Experiment

**Fig 2:**
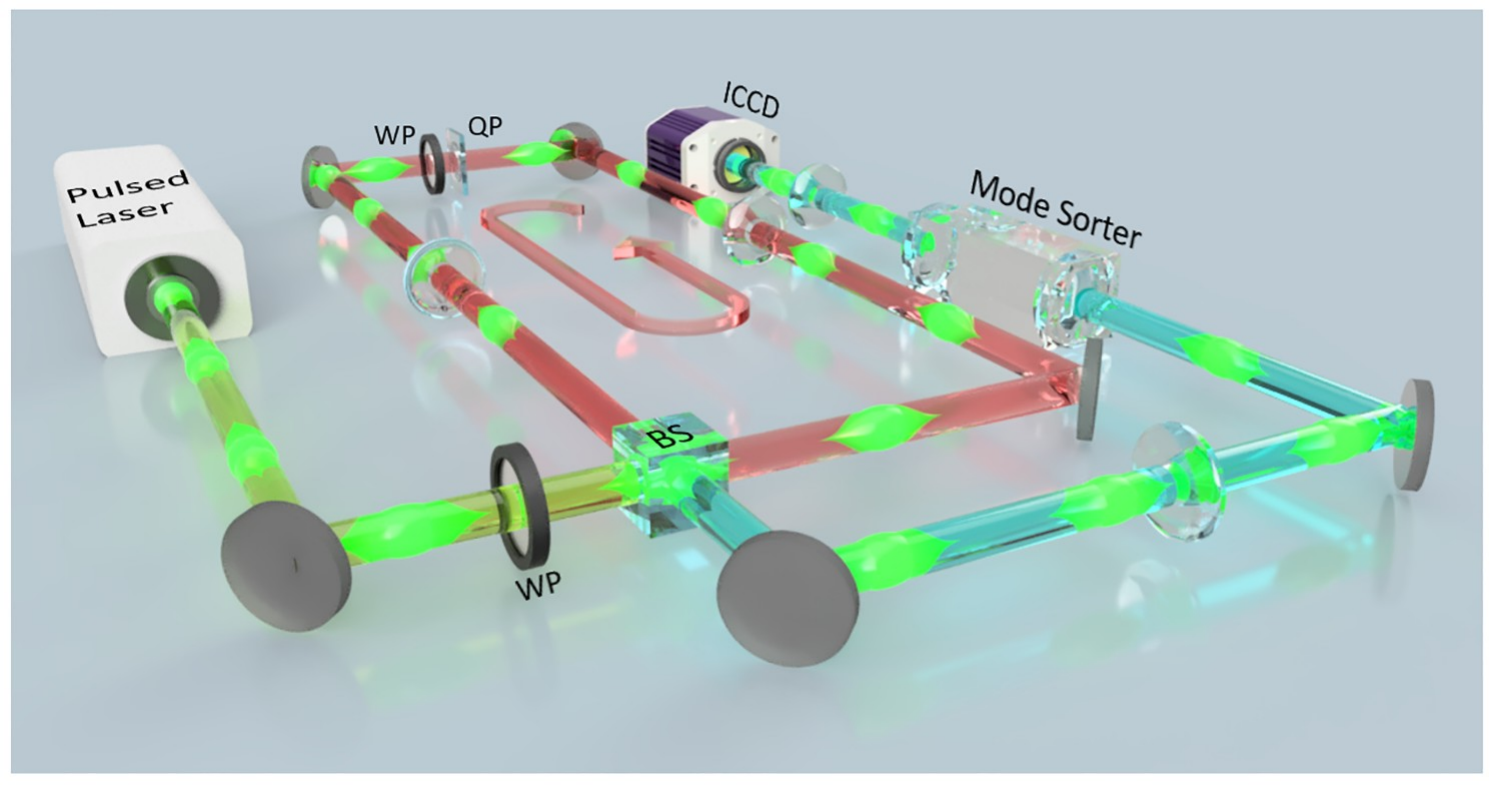
Experimental concept. A bright laser pulse with a Gaussian spatial mode is directed into a looped resonator through a beam-splitter (BS) as shown by the green path. A wave-plate (WP) initializes the state of the walker. The pulse is looped inside the cavity, highlighted by the red path in the indicated direction. Intra-cavity imaging optics to ensure that all spatial modes are divergence compensated. A q-plate (QP) and WP inside the cavity generates the step and coin toss respectively, allowing for the evolution of the walker in OAM space. After each round trip (one step of the walk), a fraction of the light is coupled out of the loop through the BS and imaged, along the blue path, onto the detection system comprising a OAM mode sorter followed by a lens, sorting the modes into transverse positions on an ICCD (Intensified Charge-Coupled Device) gated camera. This concept figure shows many pulses circulating in the resonator, however, experimentally only a single pulse is utilized. Various QWs can be implemented by adjusting the input mode, type of wave-plate and wave-plate (WP) orientation, as detailed in main text.

Following the aforementioned theory, we realize a QW using OAM as the position space and polarisation as the coin toss, within a looped resonator cavity that incorporates a *q*-plate, as illustrated in Fig 2, with further details in the Supplementary Information. The QW was initiated with a homogeneous vertically polarised (|*V*〉) Gaussian beam of pulse duration ≈ 10 ns, with the initial state determined by the angle of the HWP. This input state, separable and with 0 OAM, was admitted into the resonator (3 m length) by a beamsplitter and circulated in a 4-f imaging arrangement to mitigate mode divergence. A percentage of the pulse was then transmitted out of the resonator while the rest was reflected for another round trip. By placing a *q*-plate (QP) and WP inside the resonator, each round trip corresponds to a step in a QW, with each step in the walker measured by the partial transmission of the pulse after each round trip. It follows that the QW evolves temporally with each step at multiples of the round trip time. Consequently, as each round trip pulse is partially transmitted by the BS, the entire probability distribution for each position may be simply retrieved with a mode sorter [45] and ICCD camera, the latter for determining the step number and the former to detect the walker position in OAM space (see Supplementary Information). Clearly by restricting the ICCD exposure time, the OAM distribution of the pulse (walker) for a single step may be isolated. The setup thus allows each step to be easily monitored, unlike previous classical light implementation in waveguides [23, 24] or cascaded q-plates [33]. Moreover, the setup is scalable since the physical resources do not change with the number of steps, but rather remain constant with only one QP-QWP pair required for an entire walk of arbitrary step number.

### Experimental results

**Fig 3:**
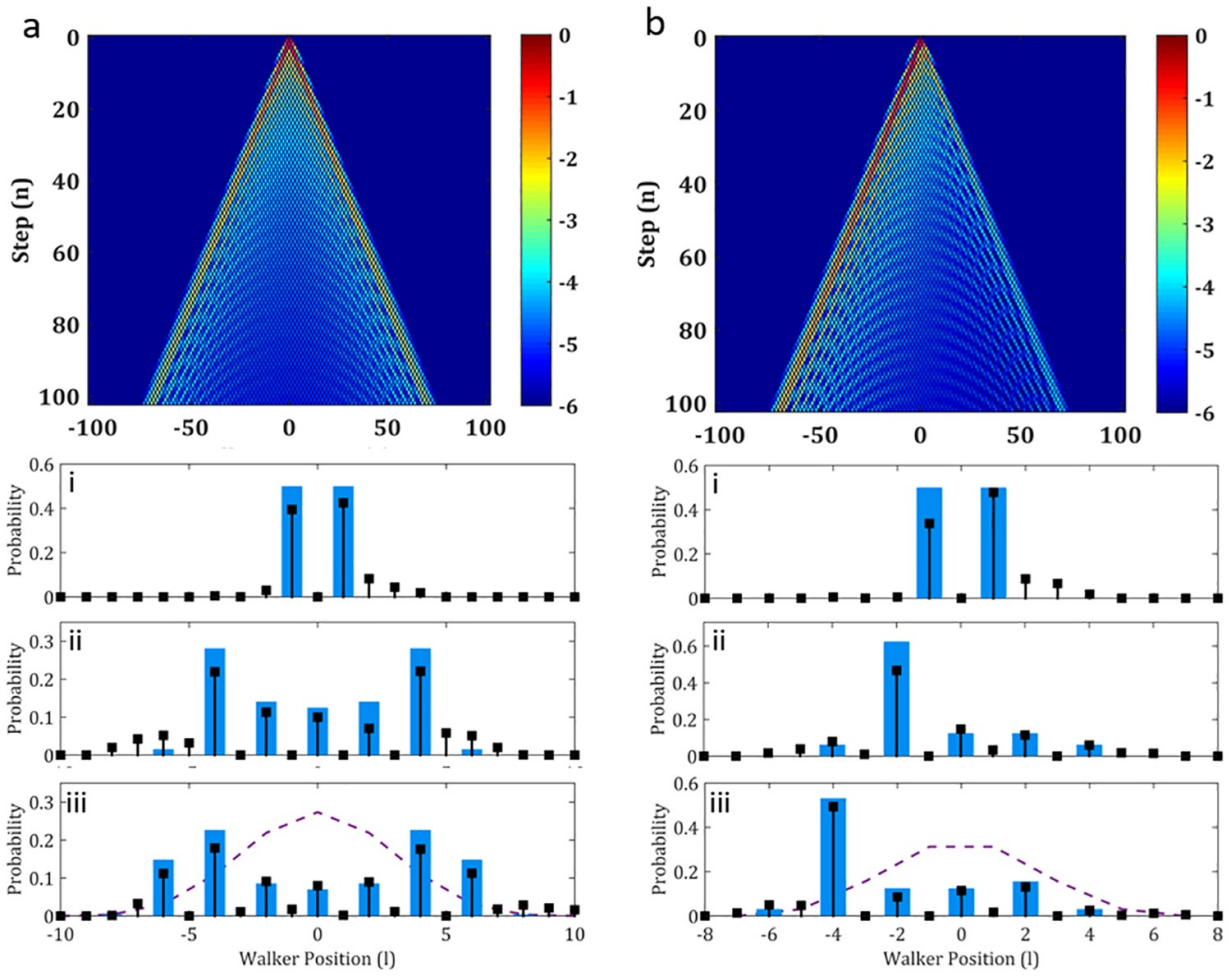
Experimentally realized Hadamard QWs. Theoretical density plots of the probability of a QW with a Hadamard coin for 100 steps with a (a) symmetrical and (b) asymmetrical input state (intensity is plotted on a logarithmic scale to emphasize the trend). Sub-plots below the density plots show the experimental realization of the walks for choice steps. Blue bars indicate the simulated probabilities and the black points the experimental data. Respectively, (i) shows Step 1 with S = 0.82 and S = 0.81, (ii) Step 6 with S = 0.80 and Step 4 with S = 0.86 and (iii) Step 8 with S = 0.85 and Step 5 with 0.90. The dashed purple line shows the classical RW distribution for comparison.

**Fig 4:**
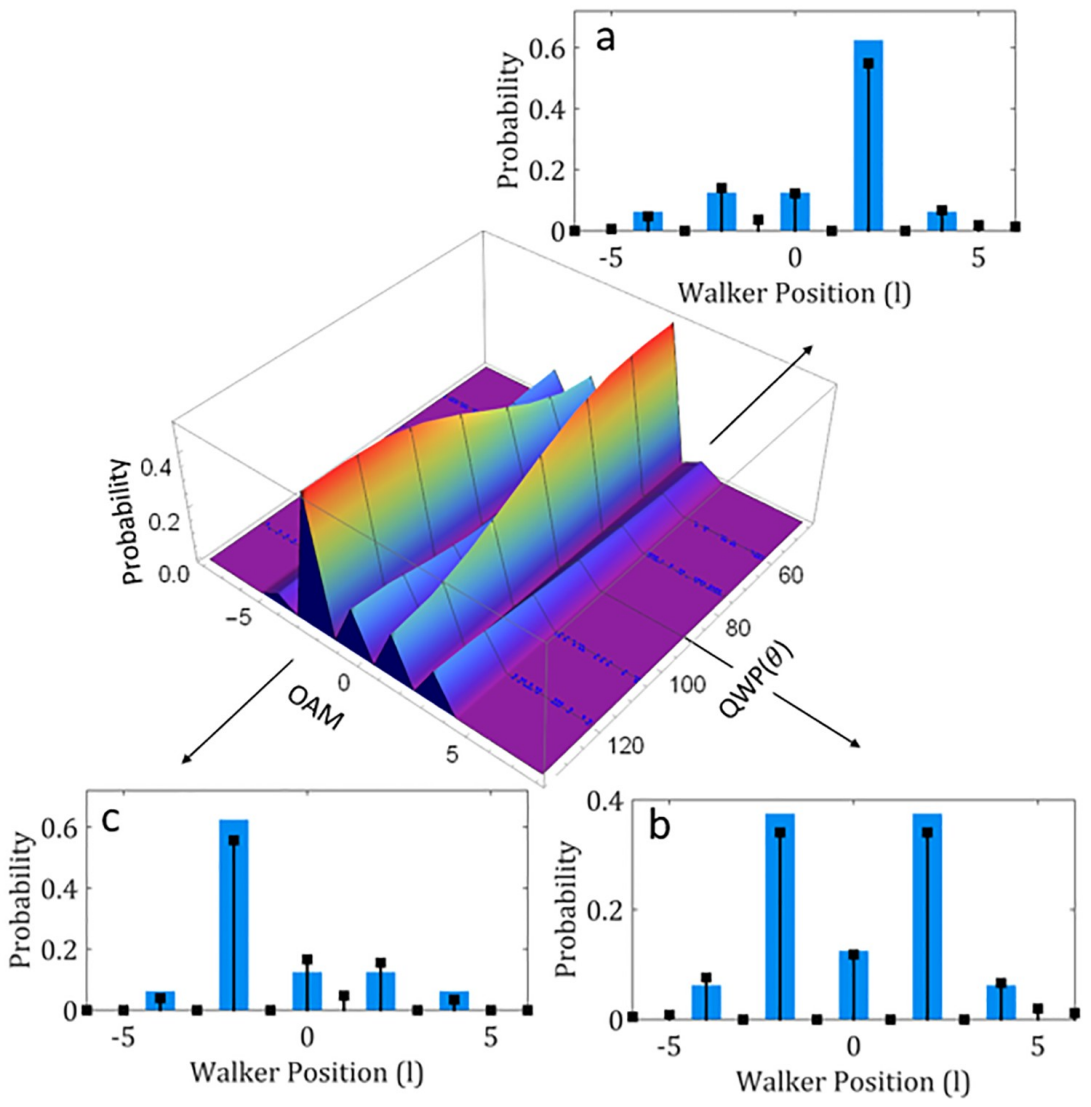
Rotating the coin. Theoretical 3D plot of change in QW symmetry with the coin angle with a QWP coin at Step 4. Insets show the theory (blue bars) and experimental data (black points) for a change in symmetry with an asymmetrical input state for the QWP at (a) 45° yielding a Hadamard coin (*S* = 0.92), (b) at 90° yielding a Balanced coin (*S* = 0.94) and (c) at 135° yielding a full reversal in the Hadamard symmetry (*S* = 0.94).

**Fig 5:**
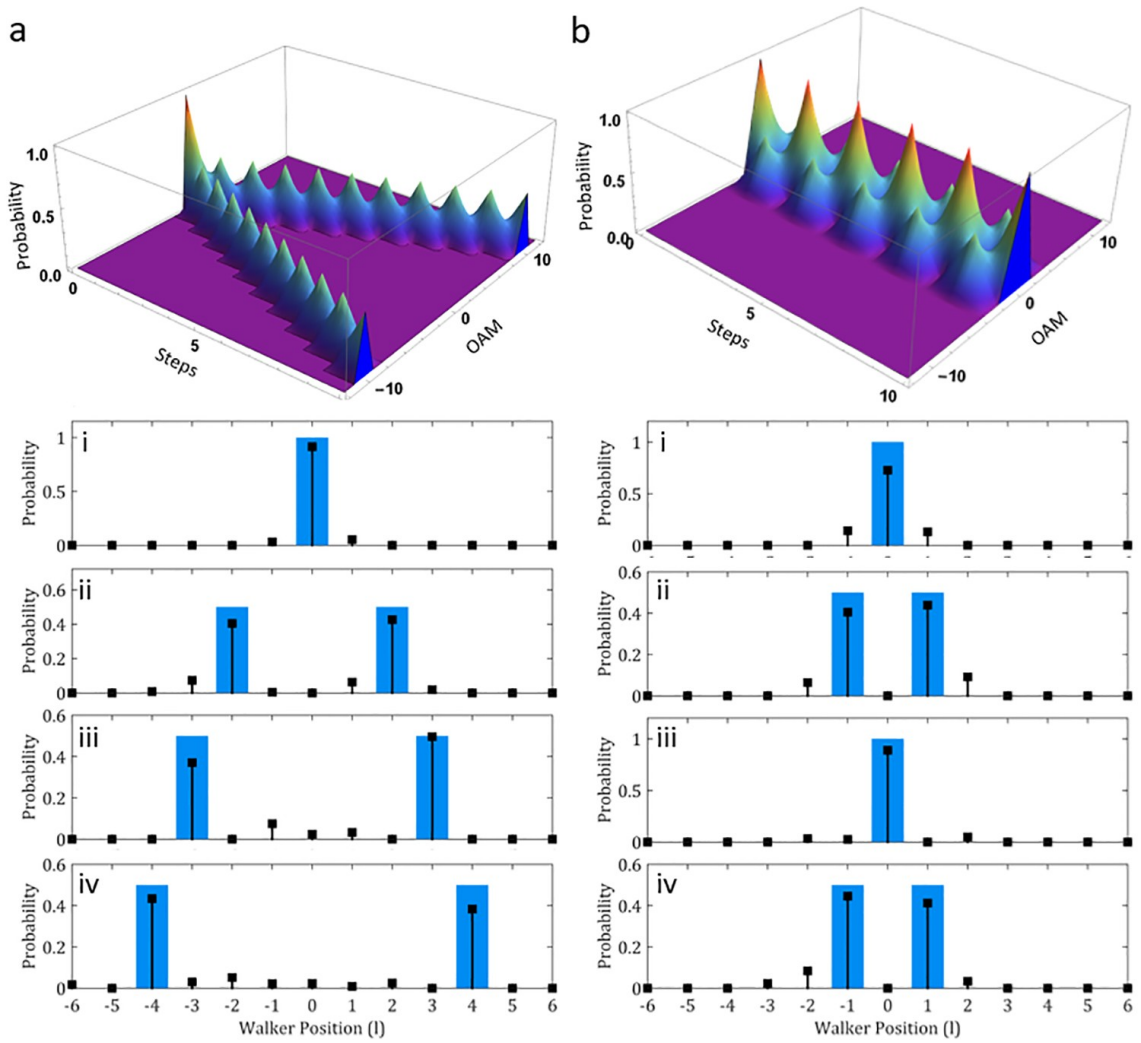
Experimentally realized QW extremes. Theoretical 3D plots of the QW probabilities for 10 steps with an (a) Identity coin and (b) NOT-coin. Sub-plots below show the experimental realization of the walks for choice steps. Blue bars indicate the simulated probabilities and the black points the experimental data. Respectively, (i) shows Step 0 with S = 0.92 and S = 0.73, (ii) Step 2 with S = 0.83 and Step 3 with S = 0.84, (iii) Step 3 with S = 0.86 and Step 4 with S = 0.89 and (iv) Step 4 with S = 0.82 and Step 5 with 0.86.

Varying combinations of initial states and coin operators were experimentally realized with the same system and subsequently shown in Figs 3, 4 and 5. Results for the Hadamard coin operator with symmetrical and asymmetrical initial states are shown in Fig 3. This was achieved by placing the QWP with the fast axis at 45° after the QP while adjusting the fast axis of the HWP before the resonator.

For a diagonal input (HWP at 67.5° acting on a vertically polarised beam) the symmetrical initial coin state evolution is shown in Fig 3(a). Here a theoretical plot of the QW evolution is given, indicating a distinct and equivalent divergence of the OAM weightings away from the central position (*ℓ* = 0) and towards the outer edges. The experimental measured distributions for Steps (i) 1, (ii) 6 and (iii) 8 are consequently shown below, displaying the telltale characteristics of a QW distribution, even though the light is purely classical. Good correlation between the measured and simulated distributions are evident with the lowest calculated Similarity (as defined in the Supplementary Information) being *S* = 0.80.

Similarly, by placing the initializing HWP at 45°, the coin state of the pulse was changed to horizontal polarization, reflecting an asymmetrical input state with respect to the Hadamard coin operator. Accordingly, the QW distribution exhibits asymmetry, as shown in Fig 3(b), where divergence of the OAM weighting away from the central position is weighted towards one (negative) direction as the walk evolves. Experimental measurements for Steps (i) 1, (ii) 4 and (iii) 5 showcase the experimental actualization of this. Starting with a similar OAM distribution as the symmetrical case at Step 1 (*S* = 0.81), which is centered around the origin, a clearly asymmetric distribution is seen by Step 4 (*S* = 0.86) which is is then further propagated at Step 5 (*S* = 0.85), in accordance with the simulated distribution. Here the asymmetrical nature of the walk is evident with the weightings detected for the OAM values towards the left being approximately 3 times greater than that of the right, alongside a distinct reduction in weightings at the central OAM value.

In both cases, a distinct difference may be seen in comparison to the classical RW distribution for the last measured step, where maximal probability is maintained at the origin with a Gaussian spread outwards.

The relative phase rotations induced by the coin operator action (and thus the waveplate) in the QW, however, also have significant impact on the symmetry of the distribution and thus how the walker propagates. This variation may be clearly seen by changing the orientation of the QWP as it alters the phase rotations between the coin states (right- and left-circular polarization) with the fast-axis angle. The theoretical 3D plot in Fig 4 illustrates this alteration from the characteristic Hadamard coin operation at 45°, yielding an asymmetrical distribution for a vertically polarized initial state to a completely symmetric distribution at 90°, forming the commonly known Balanced coin. Continued rotation of the angle then results in a reversal of the asymmetry at 135° in comparison to the Hadamard case. Experimental results are given for these extreme cases as shown by the insets graphs where good agreement occurs for the (a) Hadamard coin with a similarity of *S* = 0.92, (b) Balanced coin with a similarity of *S* = 0.94 and (c) a reversed Hadamard coin with similarity *S* = 0.94.

As the QW distribution results from the walker interference with itself, it follows that the phenomenon holds extremes in this respect where the walker either does not interfere with itself or fully interferes with itself. These extremes are enacted by the Identity and NOT-coins respectively which are illustrated in Fig 5(a) and 5(b) respectively. By inserting a HWP at 0° inside the cavity we can produce the identity coin. The 3D plot illustrates the characteristic evolution of this walk over 10 steps. Here the operation of this coin is to continually ladder each state in the same direction, causing no interference between them. As a result, this coin generates states at the extremes of the distribution for each step, such that maximal variance is seen for the QW. Experimental realization of this is shown in plots (i–iv) of Fig 5(a), where the only states occupied are the OAM values corresponding to the step number. For instance, (i) Step 0 occupies OAM 0 (S = 0.92), (ii) Step 2 occupies OAM ±2 (S = 0.83) (iii) Step 3 occupies OAM ±3 (S = 0.86) and (iv) Step 4 occupies OAM ±3 (S = 0.82).

The built-in coin resulting from the polarization flipping action of the QP naturally forms the NOT coin, causing the walker to invert polarization states and thus walker direction at each step. The walker then oscillates about the origin, perpetually propagating from *l* = 0 to ±1 and back as shown in the 3D plot of Fig 5(b). Results for this coin are shown in Fig 5(b)(i)–5(b)(iv). Here the distinct confinement of the distribution to the initial position of the walker is evident throughout the steps. Step 0 (i) (S = 0.73) and Step 4 (iii) (S = 0.89), in particular, shows the walker at the origin, baring significant contrast to the same step distributions shown for Fig 5. Steps 3 (ii) (S = 0.84) and 5 (iv) (S = 0.86) highlights the maximum spread possible for this walk with the extreme limited to ±1 for alternate steps.

## Discussion and conclusion

It is well known that classical light can be used to mimic a QW, and has been achieved by a complex arrangement of connected interferometers, often on a waveguide [30] or in a fibre [31], akin to a Galton board [29]. In this way, the quantum principle of superposition is replaced by classical interference of many paths [46]. This approach is experimentally challenging and is not scalable, requiring ever more elements for each increase in step number. Implementing a resonator architecture overcomes this issue where the position space is represented by a path-independent property of the walker, such as time-binned pulses propagating in a fibre [47]. Here the scaling potential is clearly seen with variations of the time-binning approach bringing the achievable step numbers up to 200 [22, 31, 45, 48]. A fundamental limitation to this approach is that the size of the position space relates directly to the size of the loop, where a larger QW requires ever larger loops and shorter pulse lengths.

Our experiment is based on a resonator type configuration in free space where non-separable states of light are used instead of time-binned light pulses, allowing for multiple steps in a QW to be observed with only a few elements and potentially overcoming the length dependence of the previous approach. Here we have exploited OAM and polarisation as our non-separable degrees of freedom, the former for the space in which the walker moves. A practical advantage of walking in OAM space relates to the physical size of this “position” space as all the twists and thus positions are superimposed in the same beam such that a theoretically infinite space for moving the walker exists in a single beam of light. Accordingly, our scheme has the potential to overcome the limitations associated with the time-binned approach.

Quantum walks in OAM space have been previously demonstrated using a cascade of optical elements for each step, again requiring a scale in elements for an increase in steps [26, 49]. Here we demonstrate, for the first time, the combination of a resonator with OAM walker space such that (i) it allows any number of steps to be achieved without adjustment to the experimental arrangement; and (ii) all positions in the walker space can be measured at once rather than iteratively. In our experiment, however, the step number has been limited by available resources: to fit the detector spectral range the available laser was frequency doubled, resulting in high losses and poor beam quality. Additionally, the q-plate generated OAM states excite unwanted radial modes [50] and with many passes through the q-plate, significant excitation of the higher order radial modes occur, further contributing to losses [51]. In principal, this scheme is highly scalable with the limiting factors reduced to intensity losses within the resonator, numerical aperture of the optics and OAM detection. Here, implementation of an amplifier in the resonator would eliminate losses. However, as the OAM spectrum moves to higher values with step number, the associated beam divergence and high spatial frequencies challenge the NA of the system, limiting the maximum OAM to *ℓ* ≈ 2*πR*/λ where *R* is the radius of the optic. Consequently, for a typical setup with wavelength, λ = 532 nm, and optical element radii of *R* = 50.8 mm, the containable OAM and thus QW step is estimated to be below 2 × 10^5^, i.e., finite but significantly larger than presently available with other schemes. With regards to detection, it has been demonstrated that measurement of over 40 OAM states is possible whereby the accuracy of each measured mode is over 70% [52] with the mode sorter. After 40 steps, realignment of the sorter would then be necessary to maintain precision.

In conclusion, by employing a resonator type configuration we are able to demonstrate a versatile and scalable QW using spatial modes of light in free-space, ushering in an alternative approach to future QW implementations.

## Supporting information

S1 Appendix Supplementary information. Here we provide supplementary information to the article “A versatile quantum walk resonator with bright classical light”. A more general look at quantum walks with the characteristics are provided and the details pertaining to implementing the experiment explored. We describe the experimental configuration, methods and material that were used. This includes a summary of the fundamental physics behind the pivotal optical elements (namely the q-plate and mode sorters) used to carry out the walk as well as a characterization of their physical operation. The timing and synchronization aspects of the experiment are also described along with associated corrections made to the detected data due to overlapping of the pulses occurring within the resonator configuration.
(PDF)

## References

[1] Wang J, Manouchehri K. Physical implementation of quantum walks. Springer; 2013.

[2] Aharonov Y, Davidovich L, Zagury N. Quantum random walks. Physical Review A. 1993;48(2):1687 10.1103/PhysRevA.48.1687 9909772

[3] Childs AM. Universal computation by quantum walk. Physical review letters. 2009;102(18):180501 10.1103/PhysRevLett.102.180501 19518851

[4] Childs AM, Gosset D, Webb Z. Universal computation by multiparticle quantum walk. Science. 2013;339(6121):791–794. 10.1126/science.1229957 23413349

[5] Aaronson S, Arkhipov A. The computational complexity of linear optics. In: Proceedings of the forty-third annual ACM symposium on Theory of computing. ACM; 2011. p. 333–342.

[6] Childs AM, Goldstone J. Spatial search by quantum walk. Physical Review A. 2004;70(2):022314 10.1103/PhysRevA.70.022314

[7] Shenvi N, Kempe J, Whaley KB. Quantum random-walk search algorithm. Physical Review A. 2003;67(5):052307 10.1103/PhysRevA.67.052307

[8] Sánchez-Burillo E, Duch J, Gómez-Gardenes J, Zueco D. Quantum navigation and ranking in complex networks. Scientific reports. 2012;2:605 10.1038/srep00605 22930671 PMC3428603

[9] Venegas-Andraca SE. Quantum walks: a comprehensive review. Quantum Information Processing. 2012;11(5):1015–1106. 10.1007/s11128-012-0432-5

[10] Cardano F, Massa F, Karimi E, Slussarenko S, Paparo D, de Lisio C, et al. Photonic quantum walk in a single beam with twisted light. arXiv preprint arXiv:14034857. 2014;.

[11] Schreiber A, Gábris A, Rohde PP, Laiho K, Štefaňák M, Potoček V, et al A 2D quantum walk simulation of two-particle dynamics. Science. 2012;336(6077):55–58. 10.1126/science.1218448 22403179

[12] Ambainis A. Quantum walk algorithm for element distinctness. SIAM Journal on Computing. 2007;37(1):210–239. 10.1137/S0097539705447311

[13] Yang YG, Pan QX, Sun SJ, Xu P. Novel image encryption based on quantum walks. Scientific Reports. 2015;5:7784 10.1038/srep07784 25586889 PMC4293593

[14] Vlachou C, Krawec W, Mateus P, Paunkovic N, Souto A. Quantum key distribution with quantum walks. arXiv preprint arXiv:171007979. 2017;.

[15] Mohseni M, Rebentrost P, Lloyd S, Aspuru-Guzik A. Environment-assisted quantum walks in photosynthetic energy transfer. The Journal of chemical physics. 2008;129(17):11B603 10.1063/1.3002335 19045332

[16] Ryan CA, Laforest M, Boileau JC, Laflamme R. Experimental implementation of a discrete-time quantum random walk on an NMR quantum-information processor. Physical Review A. 2005;72(6):062317 10.1103/PhysRevA.72.062317

[17] Feist A, Echternkamp KE, Schauss J, Yalunin SV, Schäfer S, Ropers C. Quantum coherent optical phase modulation in an ultrafast transmission electron microscope. Nature. 2015;521(7551):200 10.1038/nature14463 25971512

[18] Karski M, Förster L, Choi JM, Steffen A, Alt W, Meschede D, et al Quantum walk in position space with single optically trapped atoms. Science. 2009;325(5937):174–177. 10.1126/science.1174436 19589996

[19] Zähringer F, Kirchmair G, Gerritsma R, Solano E, Blatt R, Roos C. Realization of a quantum walk with one and two trapped ions. Physical review letters. 2010;104(10):100503 10.1103/PhysRevLett.104.100503 20366407

[20] Schmitz H, Matjeschk R, Schneider C, Glueckert J, Enderlein M, Huber T, et al Quantum walk of a trapped ion in phase space. Physical review letters. 2009;103(9):090504 10.1103/PhysRevLett.103.090504 19792773

[21] Alberti A, Wimberger S. Quantum walk of a Bose-Einstein condensate in the Brillouin zone. Physical Review A. 2017;96(2):023620 10.1103/PhysRevA.96.023620

[22] Schreiber A, Cassemiro K, Potoček V, Gábris A, Jex I, Silberhorn C. Decoherence and disorder in quantum walks: from ballistic spread to localization. Physical review letters. 2011;106(18):180403 10.1103/PhysRevLett.106.180403 21635071

[23] Perets HB, Lahini Y, Pozzi F, Sorel M, Morandotti R, Silberberg Y. Realization of quantum walks with negligible decoherence in waveguide lattices. Physical review letters. 2008;100(17):170506 10.1103/PhysRevLett.100.170506 18518267

[24] Sansoni L, Sciarrino F, Vallone G, Mataloni P, Crespi A, Ramponi R, et al Two-particle bosonic-fermionic quantum walk via integrated photonics. Physical review letters. 2012;108(1):010502 10.1103/PhysRevLett.108.010502 22304249

[25] Wang X, Xiao L, Qiu X, Wang K, Yi W, Xue P. Detecting topological invariants and revealing topological phase transitions in discrete-time photonic quantum walks. Physical Review A. 2018;98(1):013835 10.1103/PhysRevA.98.013835

[26] Cardano F, Massa F, Qassim H, Karimi E, Slussarenko S, Paparo D, et al Quantum walks and wavepacket dynamics on a lattice with twisted photons. Science Advances. 2015;1(2):e1500087 10.1126/sciadv.1500087 26601157 PMC4643825

[27] Cardano F, Maffei M, Massa F, Piccirillo B, De Lisio C, De Filippis G, et al Statistical moments of quantum-walk dynamics reveal topological quantum transitions. Nature Communications. 2016;7:11439 10.1038/ncomms11439 27102945 PMC4844751

[28] Gräfe M, Heilmann R, Perez-Leija A, Keil R, Dreisow F, Heinrich M, et al On-chip generation of high-order single-photon W-states. Nature Photonics. 2014;8(10):791 10.1038/nphoton.2014.204

[29] Bouwmeester D, Marzoli I, Karman GP, Schleich W, Woerdman J. Optical galton board. Physical Review A. 1999;61(1):013410 10.1103/PhysRevA.61.013410

[30] Qi F, Wang Y, Ma Q, Zheng W. Experimentally simulating quantum walks with self-collimated light. Scientific reports. 2016;6:28610 10.1038/srep28610 27353428 PMC4926089

[31] Regensburger A, Bersch C, Hinrichs B, Onishchukov G, Schreiber A, Silberhorn C, et al Photon propagation in a discrete fiber network: An interplay of coherence and losses. Physical review letters. 2011;107(23):233902 10.1103/PhysRevLett.107.233902 22182090

[32] Boutari J, Feizpour A, Barz S, Di Franco C, Kim M, Kolthammer W, et al Large scale quantum walks by means of optical fiber cavities. Journal of Optics. 2016;18(9):094007 10.1088/2040-8978/18/9/094007

[33] Cardano F, D’Errico A, Dauphin A, Maffei M, Piccirillo B, de Lisio C, et al Detection of Zak phases and topological invariants in a chiral quantum walk of twisted photons. Nature Communications. 2017;8:15516 10.1038/ncomms15516 28569741 PMC5501976

[34] Perez-Leija A, Soto-Eguibar F, Chavez-Cerda S, Szameit A, Moya-Cessa H, Christodoulides DN. Discrete-like diffraction dynamics in free space. Optics express. 2013;21(15):17951–17960. 10.1364/OE.21.017951 23938667

[35] Eichelkraut T, Vetter C, Perez-Leija A, Moya-Cessa H, Christodoulides DN, Szameit A. Coherent random walks in free space. Optica. 2014;1(4):268–271. 10.1364/OPTICA.1.000268

[36] Venegas-Andraca SE. Quantum walks for computer scientists. Synthesis Lectures on Quantum Computing. 2008;1(1):1–119. 10.2200/S00144ED1V01Y200808QMC001

[37] Goyal SK, Roux FS, Forbes A, Konrad T. Implementing quantum walks using orbital angular momentum of classical light. Physical review letters. 2013;110(26):263602 10.1103/PhysRevLett.110.263602 23848875

[38] Borges C, Hor-Meyll M, Huguenin J, Khoury A. Bell-like inequality for the spin-orbit separability of a laser beam. Physical Review A. 2010;82(3):033833 10.1103/PhysRevA.82.033833

[39] Kagalwala KH, Di Giuseppe G, Abouraddy AF, Saleh BE. Bell’s measure in classical optical coherence. Nature Photonics. 2013;7(1):72 10.1038/nphoton.2012.312

[40] Ghose P, Mukherjee A. Entanglement in classical optics. Reviews in Theoretical Science. 2014;2(4):274–288. 10.1166/rits.2014.1024

[41] Lee K, Thomas J. Entanglement with classical fields. Physical Review A. 2004;69(5):052311 10.1103/PhysRevA.69.052311 11864055

[42] Spreeuw RJ. A classical analogy of entanglement. Foundations of physics. 1998;28(3):361–374. 10.1023/A:1018703709245

[43] Simon R, Mukunda N. Minimal three-component SU (2) gadget for polarization optics. Physics Letters A. 1990;143(4-5):165–169. 10.1016/0375-9601(90)90732-4

[44] Goyal SK, Konrad T, Diósi L. Unitary equivalence of quantum walks. Physics Letters A. 2015;379(3):100–104. 10.1016/j.physleta.2014.11.001

[45] Berkhout GC, Lavery MP, Courtial J, Beijersbergen MW, Padgett MJ. Efficient sorting of orbital angular momentum states of light. Physical review letters. 2010;105(15):153601 10.1103/PhysRevLett.105.153601 21230900

[46] Knight PL, Roldán E, Sipe J. Quantum walk on the line as an interference phenomenon. Physical Review A. 2003;68(2):020301 10.1103/PhysRevA.68.020301

[47] Schreiber A, Cassemiro KN, Potoček V, Gábris A, Mosley PJ, Andersson E, et al Photons walking the line: a quantum walk with adjustable coin operations. Physical review letters. 2010;104(5):050502 10.1103/PhysRevLett.104.050502 20366754

[48] Wimmer M, Price HM, Carusotto I, Peschel U. Experimental measurement of the Berry curvature from anomalous transport. Nature Physics. 2017;13(6):545 10.1038/nphys4050

[49] Zhang P, Ren XF, Zou XB, Liu BH, Huang YF, Guo GC. Demonstration of one-dimensional quantum random walks using orbital angular momentum of photons. Physical Review A. 2007;75(5):052310 10.1103/PhysRevA.75.052310

[50] Karimi E, Zito G, Piccirillo B, Marrucci L, Santamato E. Hypergeometric-gaussian modes. Optics letters. 2007;32(21):3053–3055. 10.1364/OL.32.003053 17975594

[51] Sephton B, Dudley A, Forbes A. Revealing the radial modes in vortex beams. Applied Optics. 2016;55(28):7830–7835. 10.1364/AO.55.007830 27828012

[52] Lavery MP, Robertson DJ, Sponselli A, Courtial J, Steinhoff NK, Tyler GA, et al Efficient measurement of an optical orbital-angular-momentum spectrum comprising more than 50 states. New Journal of Physics. 2013;15(1):013024 10.1088/1367-2630/15/1/013024

